# Achieving software-equivalent accuracy for hyperdimensional computing with ferroelectric-based in-memory computing

**DOI:** 10.1038/s41598-022-23116-w

**Published:** 2022-11-10

**Authors:** Arman Kazemi, Franz Müller, Mohammad Mehdi Sharifi, Hamza Errahmouni, Gerald Gerlach, Thomas Kämpfe, Mohsen Imani, Xiaobo Sharon Hu, Michael Niemier

**Affiliations:** 1 Department of Computer Science and Engineering, University of Notre Dame, Notre Dame, IN 46556 USA; 2 Center Nanoelectronic Technologies, Fraunhofer Institute for Photonic Microsystems, An d. Bartlake 5, 01109 Dresden, Saxony Germany; 3 Department of Computer Science, University of California Irvine, 510 E Peltason Dr., Irvine, CA 92697 USA; 4 Institut für Festkörperelektronik, Technische Universität Dresden, Mommsenstraße 15, 01062 Dresden, Saxony Germany

**Keywords:** Electrical and electronic engineering, Computer science, Computational nanotechnology

## Abstract

Hyperdimensional computing (HDC) is a brain-inspired computational framework that relies on long hypervectors (HVs) for learning. In HDC, computational operations consist of simple manipulations of hypervectors and can be incredibly memory-intensive. In-memory computing (IMC) can greatly improve the efficiency of HDC by reducing data movement in the system. Most existing IMC implementations of HDC are limited to binary precision which inhibits the ability to match software-equivalent accuracies. Moreover, memory arrays used in IMC are restricted in size and cannot immediately support the direct associative search of large binary HVs (a ubiquitous operation, often over 10,000+ dimensions) required to achieve acceptable accuracies. We present a multi-bit IMC system for HDC using ferroelectric field-effect transistors (FeFETs) that simultaneously achieves software-equivalent-accuracies, reduces the dimensionality of the HDC system, and improves energy consumption by 826x and latency by 30x when compared to a GPU baseline. Furthermore, for the first time, we experimentally demonstrate multi-bit, array-level content-addressable memory (CAM) operations with FeFETs. We also present a scalable and efficient architecture based on CAMs which supports the associative search of large HVs. Furthermore, we study the effects of device, circuit, and architectural-level non-idealities on application-level accuracy with HDC.

## Introduction

Hyperdimensional computing (HDC) is an emerging neuro-inspired computational framework. HDC is based on the mathematical properties of hyper-dimensional spaces and is closely associated with cognition and perception in human memory^1^. The computational units in HDC are hyper-dimensional vectors, called hypervectors (HVs), which are (pseudo-)random, holographic vectors with identically and independently distributed (i.i.d.) elements. HDC is lightweight in nature and is suitable for power-constrained environments such as edge computing. Furthermore, HDC can learn by looking at a small number of training images as opposed to neural networks and other machine learning approaches, which is also desired for edge implementations. HDC systems are commonly comprised of an encoding module and an associative search module. The encoding module maps the input data to a high-dimensional space and the associative search module stores encoded data and considers its similarity with given queries for inference. HDC has shown promise in a wide range of applications that involve temporal patterns, such as classification^2^, signal processing^3^, robotics^4^, and cognitive computing^5^. HDC has achieved similar or better accuracies compared to convolutional neural networks^4^, support vector machines^3^, and gradient boosting^6^ for the aforementioned applications.

HDC is particularly amenable to in-memory computing (IMC) implementations using (emerging) memory technologies as: (i) HDC performs basic element-wise operations on HVs, such as binding, bundling, and permutation to compute and learn that can greatly benefit from the parallelism offered by IMC; (ii) due to the holographic nature of HVs, no HV element is more important than any other, which improves resilience to errors and variations^7^ (especially important when considering IMC architectures based on emerging devices); (iii) HDC can work with low precision HV elements which is (at least at present) more commonplace with IMC realizations. Indeed, representative work in the literature has considered designs, demonstrations, and investigations of the viability of IMC for HDC using traditional CMOS technology, as well as emerging technologies such as resistive random access memories (RRAMs)^8–12^ and phase-change memories (PCMs)^13^.

Although IMC is an excellent candidate to accelerate HDC, there are still challenges that need to be addressed. First, it is imperative that energy, latency, and area improvements of IMC implementations do not come at the cost of accuracy loss^14^. While binary IMC implementations are highly efficient, they suffer from accuracy loss compared to software implementations running on traditional computers^15^. This represents a likely impediment when considering the adoption/deployment of HDC solutions. To compensate for low precision elements, binary IMC solutions often operate on HVs with more than 10k elements which can produce more acceptable - although still not software-equivalent—accuracies^15^. Moreover, larger HVs diminish the net energy and latency improvements derived from IMC. Finally, existing IMC HDC implementations rely heavily on ternary content-addressable memories (TCAMs) for Hamming distance measurements^8,11^ or crossbar arrays for binary dot-product operations^13^, i.e., to approximate the cosine distance function commonly employed in HDC algorithms. As such, fast and energy efficient multi-bit circuit primitives for comparison of HVs are imperative for a path to software-equivalent accuracies.

Second, scalable and realistic architectures must be developed to support operations on long HVs. Memory arrays used to store HVs and compute with them are fundamentally limited in size due to considerations like IR-drop^16^, sensing^17^, etc. Although this might be manageable for encoding - which mainly consists of independent computations for each dimension of HVs - it is much more challenging for associative search that must accumulate information across HV dimensions^17^. Previous work has considered the accumulation aspect of associative search in two different ways: (i) work in^11^ assumed it to be possible to store all HVs in a single array (optimistic as will be seen), and (ii) work in^8,12,13^ used multiple subarrays to aggregate the results in the digital or analog domain. The latter introduces new challenges as aggregating results in the analog domain can be quite susceptible to noise^8^ and requires the ability to drive large amounts of current from subarrays to the periphery^12^. Aggregation in the digital domain is more realistic, but has been mainly explored using expensive analog-to-digital converters (ADCs)^13^. A recent PCM demonstration partitioned a large array and stored HVs on multiple rows which is in principle akin to using multiple sub-arrays^13^.

We consider the use of ferroelectric field-effect transistor (FeFETs)^18^ to implement an HDC architecture and improve the efficiency of HDC algorithms, all while charting a path to iso-accuracy versus a software-based solution. FeFETs have attracted interest since the discovery of ferroelectricity in hafnium oxide^19^, which enabled CMOS-compatible realizations of FeFET devices^20^. FeFETs are non-volatile three-terminal devices, have high I$_{\text{on}}$/I$_{\text{off}}$ ratios, and allow for a wide range of circuit designs such as crossbar arrays^21^, CAMs^22–24^, oscillators^25^, frequency multipliers^26^, and functional hardware obfuscation^27^. FeFETs have been used to accelerate deep neural networks (DNNs)^21,28,29^, few-shot learning^23,30,31^, spiking neural networks (SNNs)^32^, and neural sampling machines^33^. Ferroelectric plasticity allows FeFETs to store multiple states via partial polarization switching and enables multi-bit computation^20^. Although there are single-cell multi-bit demonstrations of 2-8 bits in the literature^21,24,29,34,35^, array-level demonstrations of multi-bit programming and computation remain a challenge.

To address the aforementioned challenges, we adopt a cross-layer design perspective^14^. Our work is motivated from a top-down perspective, but our solution is best presented from a bottom-up perspective which inspires bi-directional cross-layer design and analysis. Specifically, (i) we experimentally demonstrate, for the first time, multi-bit array-level programming and computation with FeFETs in the form of multi-bit content-addressable memories^31^. We demonstrate in-situ parallel similarity search based on the squared Euclidean distance function. (ii) We propose a high-dimensional scalable CAM-based architecture for associative search that uses low-power, voltage-based sense-amplifiers (SAs) and voting to aggregate results between sub-arrays. (iii) We propose an FeFET IMC solution for HDC where we use multi-bit crossbar arrays for matrix-vector multiplication (MVM) during encoding, and multi-bit CAMs (MCAMs) for associative search. These allow us to surmount the limitations of binary implementations, and achieve iso-accuracy with respect to software implementations, all while delivering further energy and latency gains compared to binary implementations. (iv) We study the impact of errors and non-idealities from different design layers, including device-to-device variation^36^, SA limitations, and voting, on the application-level accuracy of different datasets that are commonly used in the HDC literature.

## Results

### FeFET MCAM concept

**Figure 1:**
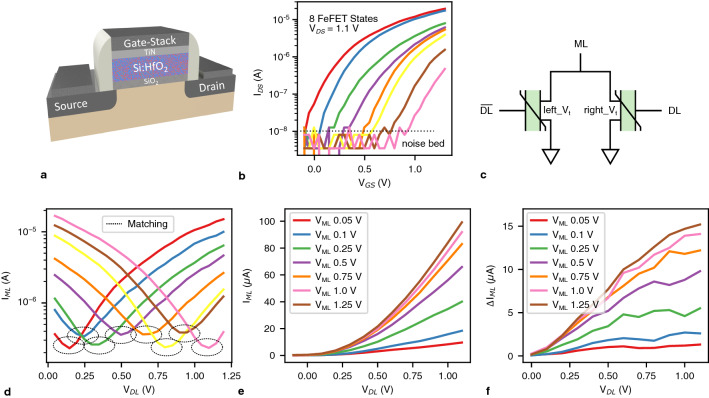
(**a**) Illustration of FeFET device concept where a FE layer is incorporated in the gate stack of a MOSFET. (**b**) Measurement results of an FeFET device in an AND array programmed to 8 distinct states. (**c**) Diagram of the universal 2FeFET CAM cell. (**d**) Measurement results of a 2FeFET MCAM cell in an AND array programmed to 8 distinct states. (**e**) Measured distance function ($I_{ML}$ vs. $V_{DL}$) of the 2FeFET MCAM for different *ML* voltages. (**f**) Differential of the 2FeFET MCAM distance function showing quadratic behavior for higher *ML* voltages and linear behavior for lower *ML* voltages.

FeFETs are realized by integrating a ferroelectric (FE) layer (usually hafnium oxide) on top of an interface layer in the gate stack of a metal-oxide-semiconductor field-effect transistor (MOSFET), resulting in a metal-ferroelectric-insulator-semiconductor (MFIS) structure^20^ (Fig. 1a). The non-volatile polarization orientation of FE domains in the FE layer impact the threshold voltage ($V_t$) of the underlying MOSFET. Polarization switching is the process of reorienting the polarization of FE domains in the FE layer by applying voltage pulses to the gate of the FeFET, which allows setting and resetting an FeFET’s $V_t$. Typically, FeFETs represent ‘0’ and ‘1’ in high $V_t$ and low $V_t$ states, respectively. As shown recently, accumulative pulse schemes can be used to gradually transition from a high $V_t$ state to a low $V_t$ state and vice versa, enabling multi-bit operation of FeFETs^21^. The state transitions in MFIS structures are defined by current percolation paths (CPPs) formed in the transistor channel^37^. Therefore, the microstructure of the FE layer plays a major role in controlling multi-bit operations in scaled FeFETs^38^ and material stack optimization is necessary for achieving desired multi-bit behavior^39^. Figure 1b demonstrates the measured data for multi-bit programming of an FeFET device to 8 states (3 bits). Multi-bit programming of FeFETs is highly desired as it enables higher density memory designs.

FeFETs enable the most compact content-addressable memory (CAM) design^40^ as they both serve as the selector and the non-volatile memory element. At the cell level, Fig. 1c shows the universal 2FeFET CAM design that can act as a ternary^30^, multi-bit^31^, or analog^41^ CAM depending on the programming and input scheme. To program this design, voltage pulses are applied to dataline (*DL*) and $\overline{DL}$ which tune the $V_t$ of the FeFETs. During search, the matchline (*ML*) is pre-charged and the input patterns are applied to *DL* and $\overline{DL}$. If the *ML* stays high, there is a match, and if *ML* discharges, there is a mismatch. In a CAM row, multiple cells are connected via the *ML* and all cells must match for the *ML* to stay high and report a match; if any cell observes a mismatch, the *ML* is discharged and the row reports a mismatch. This is called exact match search^17^ as the input pattern must exactly match the pattern stored on the CAM.

To program the 2FeFET CAM as a *b*-bit MCAM, we need $2^b$ distinct FeFET $V_t$ values $\{V_{t1}, V_{t2}, ..., V_{t2^b}\}$. To program the cell to state *s* where $s \in \{1, 2, ..., 2^b\}$, the $right\_ V_t$ (Fig. 1c) is programmed to $V_{ts}$ and the $left\_ V_t$ (Fig. 1c) is programmed to $V_{t(2^b-s+1)}$. As such, we can store $2^b$ non-overlapping ranges where each range is a state (Supplementary Fig. 1). For example, to program a 3-bit MCAM cell to state 3, the right and left $V_t$ are programmed to $V_{t3}$ and $V_{t6}$, respectively. Similarly, to search the 2FeFET CAM as a *b*-bit MCAM, we need $2^b$ distinct search voltage values $\{V_1, V_2, ..., V_{2^b}\}$. To search for input state *s* where $s \in \{1, 2, ..., 2^b\}$, $V_{DL}$ and $V_{\overline{DL}}$ (Fig. 1c) are set to $V_s$ and $V_{(2^b-s+1)}$, respectively. For example, to search for state 3 in a 3-bit MCAM, $V_{DL}$ and $V_{\overline{DL}}$ are set to $V_3$ and $V_6$, respectively. This programming and input scheme is based on the analog inverse principle^42,43^ where for any *s*, $right\_ V_t + left\_ V_t = V_{t1} + V_{t2^b} = 2 * V_{center}$ and $V_{DL} + V_{\overline{DL}} = V_1 + V_{2^b} = 2 * V_{center}$ where *center* is the analog center. Supplementary Fig. 1 shows the $V_t$ and $V_{DL}$ values for our 2- and 3-bit implementations and the examples in this paragraph.

Figure 1d shows the measured *ML* current of a 3-bit MCAM cell for different cell states. We use FeFET AND memory arrays^44^ where the equivalent MCAM cell (Fig. 1c) is constructed by using two FeFETs which are connected along their drain contacts, while their source contacts are connected to ground (Supplementary Fig. 2). *DL* voltage is swept, and the $\overline{DL}$ voltage is determined by $V_{DL} + V_{\overline{DL}} = 1.1V$ (analog inverse). For each cell state (different colors), the *ML* current should be lowest when there is a match; this represents how the exact match search works. The current of the MCAM cell differentiates between the different degrees of mismatches. Fig. 1d suggests that the further away the input is from the matching range, the higher the *ML* current of the cell. As such, the *ML* current is a function of the distance between the cell state and the input state, and can be employed to compute a distance function^31^. In this design, the distance function is effectively the FeFET transfer characteristic ($I_D - V_{GS}$ curve) as only one of the FeFETs in the cell is “ON” when there is a mismatch^31^, which in turn determines the *ML* current. As such, the behavior of the distance function depends on the operation regime of the FeFET (linear or saturation) which in turn depends on the *ML* voltage^43^. FeFETs follow field-effect transistor characteristics, therefore:

$$\begin{aligned} V_{GS} - V_t> V_{DS}= & {} gt; I_D \approx \beta *(V_{GS} - V_t)*V_{DS} ~(linear ~regime) \\ V_{GS} - V_t < V_{DS}&=> I_D \approx \beta /2*(V_{GS} - V_t)^2*V_{DS} ~(saturation ~regime) \end{aligned}$$

where $V_{GS}$ is the gate-source voltage, $V_{DS}$ is the drain-source voltage, and $I_D$ is the drain current. The FeFET drain current has a linear relation with $V_{GS} - V_t$ in the linear regime and a quadratic relation in the saturation regime. In the MCAM cell, $V_{GS}$ is determined by the input state ($V_{DL}$ and $V_{\overline{DL}}$), $V_t$ is determined by the cell state ($right\_ V_t$ and $left\_ V_t$), and $V_{DS}$ and $I_D$ are the *ML* voltage and current, respectively. Thus, the *ML* voltage determines the linear or quadratic behavior of the distance function, and the input and cell states determine the *ML* current. (Note that these characteristics may be slightly different for highly scaled devices due to short channel effects^45^, but can still realize useful distance functions^42^).

Figure 1e and f show the measured transfer characteristics of a FeFET (distance function) and its differential with different *ML* voltages, respectively. As expected, with lower *ML* voltages, the differential of the distance function is a horizontal line which indicates a linear relation between input voltage and *ML* current. For higher *ML* voltages, the differential is linear which indicates a quadratic relation between input voltage and *ML* current. For 0.25 V and 0.5 V *ML* voltages, the differential is initially linear and then turns into a horizontal line effectively transitioning from the saturation regime to the linear regime. As such, the FeFET MCAM cell can support three types of distance functions, namely, linear (Manhattan), quadratic (squared Euclidean), and a quadratic-linear combination which was previously termed as a sigmoid-like distance function^42^. As we will show, MCAM distance functions can facilitate more intricate search operations than the traditionally used exact match search^17^. In this paper, we focus on the squared Euclidean distance function (which is interchangeable with Euclidean for comparison of data^46^) as it is widely used and achieves state-of-the-art accuracies ^47,48^.

In an MCAM row, the current contribution of each CAM cell is added along the *ML* based on Ohm’s law^17^. Thus, the total *ML* current of an MCAM row represents the similarity of the pattern stored on it with the query pattern, based on the distance function. When searching an MCAM array, we can measure the similarity of query with multiple patterns in O(1) time. Finding the *ML* with the lowest current, which should represent the pattern most similar to the query or its nearest neighbor, and is called a best match search^17^. To do this, it is possible to directly convert the *ML* currents to digital values via analog-to-digital converters (ADCs) and compare them to find the lowest value^13^, although ADCs introduce significant energy and area overheads. Another approach is to pre-charge the *ML* and sense the discharge rate of the *ML* with SAs which, as we will show, is much less expensive than ADCs.

### FeFET MCAM demonstration

For the first time, we experimentally demonstrate best match search with FeFET MCAMs. This includes multi-bit programming of FeFET arrays and parallel search among multiple *ML*s and *DL*s. The MCAMs are constructed from FeFET AND arrays (see Methods). To the best of our knowledge, previous demonstrations of FeFET CAMs are limited to binary precision^23^ or single cell measurements^24^. Unlike single cell programming of FeFETs, array-level control of FeFETs is particularly challenging due to write disturbances^49^. Programming multiple states further exacerbates the challenges as the target $V_t$ values will be in closer proximity to each other, and disturbances and device-to-device variations^36,50^ are more likely to lead to programming errors. Moreover, smaller FeFET devices are harder to program due to a smaller number of domains in the FE layer^36^. The FeFETs used in our demonstration have a channel width and length of 450 nm and 450 nm, respectively, and are the smallest devices demonstrated for multi-bit array-level programming in the literature. As such, we demonstrate array-level programming and single-step parallel search for a 2-bit MCAM, while our 3-bit demonstrations are for single-cell programming. We characterize three AND arrays to which we will refer to as MCAMs (MCAM 1, MCAM 2, and MCAM 3) in the remainder of this section.

**Figure 2:**
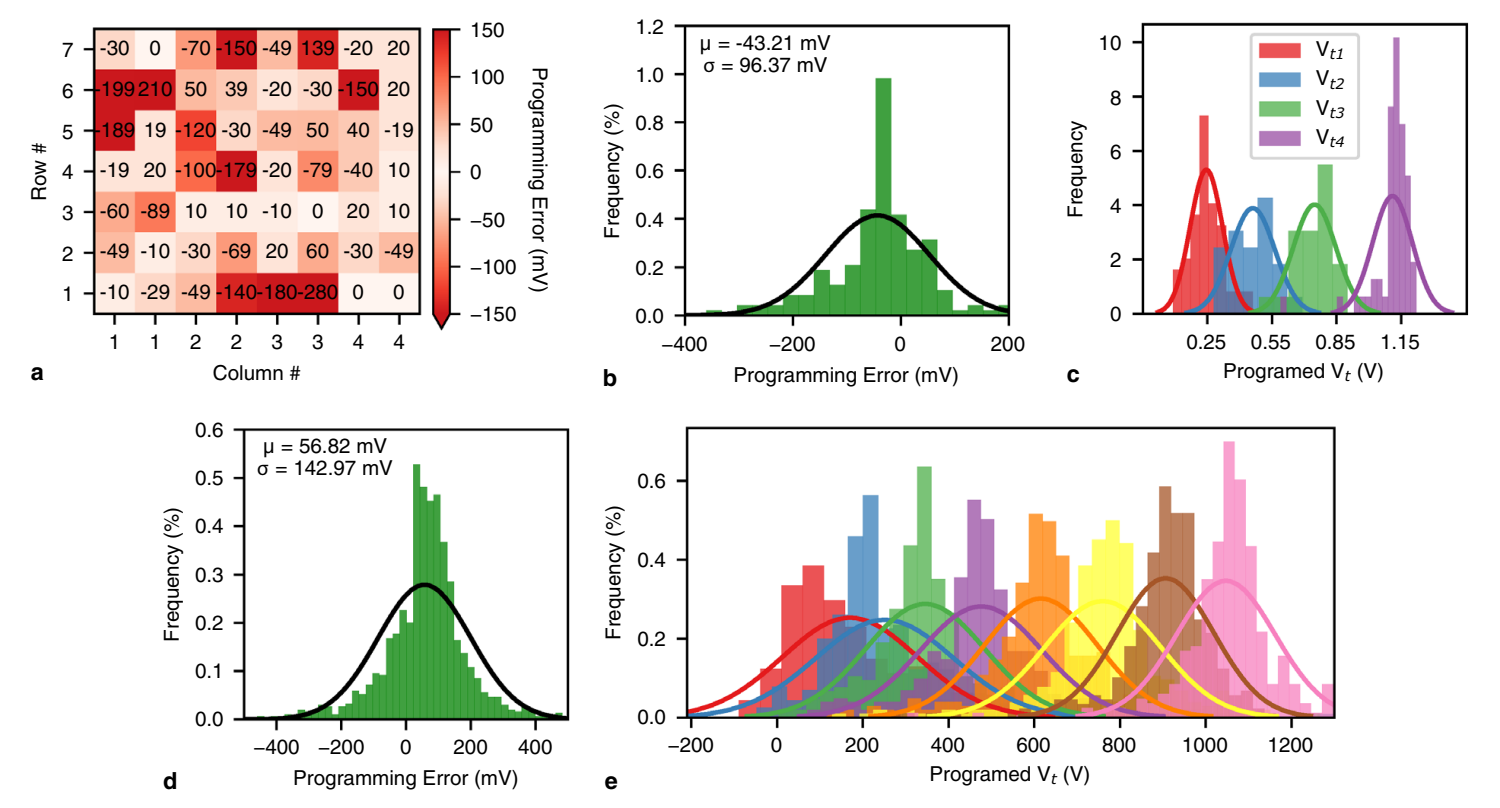
(**a**) Shows the difference between the programmed $V_t$ and the target $V_t$ (programming error) for MCAM 1 with 2-bit precision. (**b**) shows the distribution of programming errors for all MCAMs with 2-bit precision. (**c**) shows the distribution of programming errors for all the MCAMs with respect to different target $V_t$s. (**d**) shows the distribution of programming errors for 1500 FeFETs with 3-bit precision. (**e**) shows the distribution of programming errors for all MCAMs with respect to different target $V_t$s.

We consider HDC applications for 2-bit MCAM demonstrations and generate seven near-orthogonal (in Euclidean space) 4-dimensional patterns to store on the MCAMs. Supplementary Table 1 shows the patterns stored on the MCAMs in both integer values (0–3) and target values of the $left\_ V_t$ and $right\_ V_t$ (Fig. 1c). A detailed description of MCAM programming is presented in Methods. Fig. 2a and Supplementary Fig. 3a–c show the difference between the programmed $V_t$s and the target $V_t$s (called programming error) for MCAM 1 and all MCAMs, respectively. We are able to program most of the FeFETs to within 100mV of their target $V_t$, although there are some devices that observe more significant programming errors. These errors are due to the limited number of domains in scaled FeFETs, which define the formation of CPPs in the transistor channel. Thus, the probability exists that a wide percolation path is abruptly formed, resulting in a sudden $V_t$ change. This behavior is accompanied by device-to-device variations in the switching process and a unidirectional write scheme which makes the FeFETs susceptible to target $V_t$ overshooting. The programming errors can be further mitigated by using more sophisticated write-verify schemes as described in^36^ and that are not readily implementable given our current experimental setup.

Figure 2b shows the distribution of the programming errors for all three arrays. Individual array distributions are shown in Supplementary Fig. 3d–f. Results show a negatively skewed mean for the distributions and outliers primarily with negative programming error. This is because programming operations gradually transition a device’s $V_t$ from high to low, and stop when the $V_t$ is lower than the target. Due to the experiment setup, writing FeFET states is solely done over the DLs, while inhibiting FeFETs that share the active DLs is solely done over the MLs and SLs. With this write scheme, write pulses are limited to only positive voltages to avoid body-diode leakage currents in the p-n-junctions of the FeFETs which would occur when utilizing negative inhibit voltages. Therefore, when inhibit voltages have to be applied, $V_t$ states can only be set from high to low and not in reverse direction. It is possible to utilize a source/drain erase scheme^51^ to implement more sophisticated write-verify schemes^36^ to correct the devices programmed to a much lower $V_t$ than the target, but as noted above, our current experiment setup does not allow such implementations. There are also some outliers with positive programming errors, since shortly after a FeFET is programmed, relaxation effects and charge detrapping can lead to partial backswitching of domains, impacting CPPs and therefore increasing $V_t$. As expected, there are variations in the programming and the standard deviations of the distributions reflect this quantitatively.

Previous single device measurements with larger devices^24^ have reported better standard deviations since the device sizes used are larger and their measurements are not affected by array-level write disturbances. As such, our results reflect a more realistic path toward scaling and array-level control for multi-bit FeFETs. Figure 2c shows the distribution of the programming errors for all three arrays with respect to the different $V_t$ targets. Individual array distributions are shown in Supplementary Fig. 3d–j. Common single-pulse write schemes that choose a fixed write voltage for each state typically show much sharper distributions for the fully programmed and fully erased states than for the intermediate states^24^. Although the lowest and highest $V_t$ states show a slightly sharper distribution, our results show little differences in programming error with respect to different $V_t$ states because: (i) better control of inner states is possible with an iterative write-verify scheme, (ii) the low $V_t$ state is more susceptible to charge trapping because it is set with the highest write voltages and the highest number of write pulses, and (iii) the write process starts from the highest $V_t$ state which exposes the devices with high $V_t$ targets to the largest number of passive inhibit pulses, and thus increases the probability of an accumulated disturb. It should be noted that the devices under study here are square-shaped state-of-the-art FeFET devices. It is to be expected that with FeFETs optimized for multi-level operation, e.g. by considering CPP formation in conjunction with microstructure-dependent width/length scaling optimization, programming errors could be further reduced. Recent observations in stack optimization could also be incorporated. It was shown that multi-level capability can be further increased by using laminated HfO2 layers^52^ or by avoiding CPP influences when using a MFMIS structure^53^.

After programming the MCAMs, we perform parallel best match search. We generate 7 queries per pattern stored on the MCAMs for a total of 49 patterns per MCAM. To generate the 7 queries, we start from the stored pattern and modify it to increase the squared Euclidean distance. Supplementary Table 2 shows the 7 queries generated based on the pattern stored on the first *ML* in Supplementary Table 1. The generation of the other queries follows the same method. To search the MCAMs, the queries are applied to the *DL*s and the *ML* currents are measured. The *ML* with the lowest current is reported as the best match for each of the 3 MCAMs. The three MCAMs correctly decide the best match for 37, 43, and 43, of the queries, respectively, for an aggregate accuracy of 84%. Although there is a general correlation between the magnitude of variations and correct search output, the specific location of the variations, queries, and stored patterns affect the output as well. For example, Fig. 2a shows that the cell in row 1 and column 3 has observed significant negative $V_t$ shift for both FeFETs. This results in high *ML* current contribution from this cell for a variety of inputs. Given the specific inputs and the specific variations in the programming of the MCAMs, MCAM 1 with the lowest magnitude of variations achieved lower accuracy than MCAM 2 and MCAM 3 with higher magnitudes of variations. Further, given that the patterns only have 4 dimensions, erroneous contributions of each cell can significantly impact the similarity of the patterns. In contrast, patterns with larger dimensions are more robust to errors (a main reason for robustness to errors in HDC). Overall, given the magnitude of variations, the small number of pattern dimensions, experiment setup limitations, and the material stack of the measured devices, the achieved demonstration accuracy is acceptable.

We also demonstrate single-cell programming of FeFETs for a 3-bit precision using a write-verify without inhibit scheme (see Methods). Figure 2d shows the distribution of 3-bit programming errors for 1500 FeFETs. The results show a higher standard deviation compared to the 2-bit results in Fig. 2b due to the following: (i) The target $V_t$ values are closer to each other (150 mV vs. 300 mV) and it is not guaranteed to be able to achieve every target $V_t$ due to the limited number of available domains. (ii) The programming $V_t$ range is larger (1050 mV vs. 900 mV) which makes it harder for devices to be programmed to the lowest $V_t$ targets. (iii) As FeFETs are programmed in arrays, when programming an MCAM cell and no inhibit scheme is utilized, all other FeFETs on the same *DL* are not voltage protected and therefore stressed/hole-trapped, which is potentially more destructive that the disturbance effects when programming an array in parallel. Further, the mean of the distribution for 3-bit results is positive whereas the 2-bit results show a negative mean. This is due to the difference in programming scheme, since in 3-bit single-cell programming, FeFETs are initially programmed to a low $V_t$ state and then gradually programmed to higher $V_t$ states with negative voltage pulses.

Figure 2e shows the achieved $V_t$’s with respect to the 8 target states. As explained above, the $V_t$ range is chosen larger compared to the 2-bit-case to accommodate all 8 states. The low $V_t$ state has the widest distributions because a single pulse is used to program the FeFETs to the lowest $V_t$, and due to device-to-device variations, the $V_t$ might not be reachable for all FeFETs. Similar to our 2-bit results, due to the better control over the intermediate states by utilizing a write-verify scheme, we can achieve tighter distributions for the intermediate states. The high $V_t$ states show the sharpest distributions, which indicates that we are operating in the sweet spot region where (i) the target $V_t$ value can be achievable by most FeFETs, (ii) we are in a switching range where the tendency to overshoot decreases, and (iii) the more gradual switching when erasing allows the state to be set more reliably.

### CAM-based architecture for associative search

CAMs can perform fast and energy efficient search operations, but are practically limited in terms of the number of dimensions they can store and search. Although CAMs (primarily TCAMs) are mainly used in network routing^54^, they have recently gained attraction and showed prominence for similarity search and attention mechanisms^55^ in machine learning applications such as few-shot learning^30,42,56^ and hyperdimensional computing^8,13,57^. These applications require high-dimensional search support, especially when solving complex problems/datasets. Thus, scalable and realistic architectures must be devised to support high-dimensional search with CAMs. Given that increasing the number of CAM dimensions (i.e., the number of columns in an array) faces physical challenges such as IR-drop^16^, sensing^17^, etc., the need to aggregate results from multiple sub-arrays in the analog or digital domain, is inevitable. While prior efforts aim to aggregate results in the analog domain^8,12^, we consider aggregation in the digital domain as we can save energy and be more robust to noise and variations.

Recent work proposed using time-domain circuits for aggregating HDC associative search results between RRAM-based CAM sub-arrays^8^ where each sub-array holds 16 dimensions of the class HVs. Each sub-array has a voltage-to-time converter which converts the number of matched cells to a time signal. The time signals from different sub-arrays are then added with a time-to-voltage converter to obtain the total number of matching cells for each class HV. That said, the simulation results presented only show the addition of time signals from two sub-arrays, whereas the paper suggests that to achieve 1k dimensions, the addition of 64 time signals is required. As mentioned by the authors of the paper^8^, this approach can be highly susceptible to noise and is limited in scalability. Another approach uses ADCs to count the number of mismatches and adds the results across different slices of binary HVs^13^. ADCs can have significant energy and area overhead and often dominate total energy and area^58^. In this work, a 512x2048 PCM memory array is employed where the array is partitioned into ‘f’ slices and holds 10,000-dimensional HVs. This is similar to having multiple sub-arrays as the search query for each partition is different, and ‘f’ steps are required to search all 10k dimensions of the HVs. Further, they face structural non-idealities in programming the PCM array due to the large array size which has negative effects on application-level accuracy^13^.

**Figure 3:**
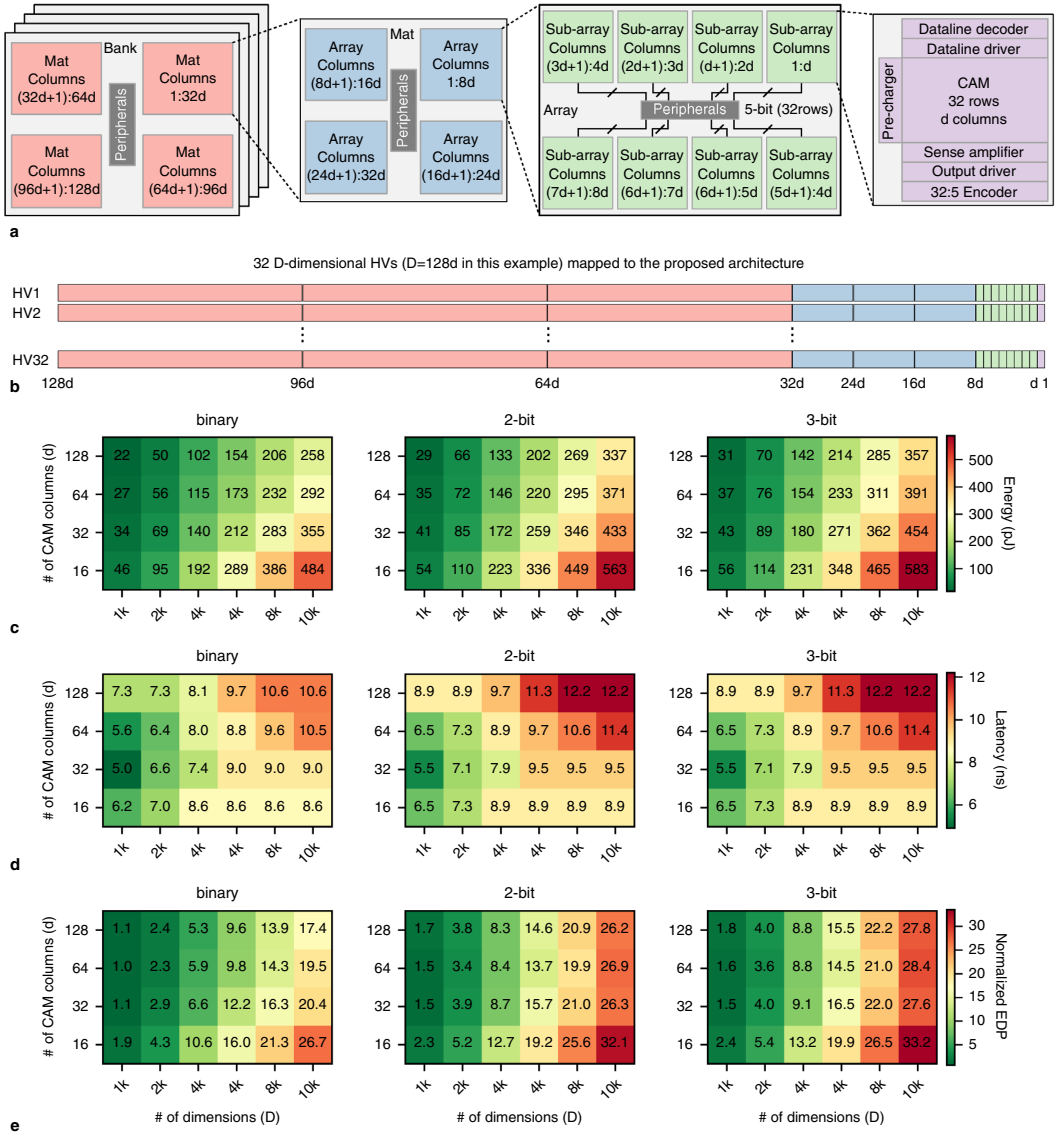
(**a**) proposed CAM-based architecture for associative search where each sub-array stores *d* dimensions of the HVs and sub-arrays are arranged in Arrays, Mats, and Banks. (**b**) an example of mapping 32 *D*-dimensional HVs to the proposed architecture where the colors of HV slices correspond to the colors of the architecture. (**c**) energy, (**d**) latency, and (**e**) normalized EDP of a single search with the proposed architecture for SA-based implementations.

Here, we propose a tiled CAM architecture that can support high-dimensional associative search via voting. Figure 3a shows the proposed architecture where 8 sub-arrays are tiled together to create an ‘Array’, 4 Arrays create a ‘Mat’, and 4 Mats create a ‘Bank’. This architecture is scalable as it can increase the number of Banks to support more dimensions. To use the proposed architecture for HDC, each sub-array holds *d* dimensions of *D*-dimensional class HVs, and up-to 32 class HVs (32 rows in each sub-array), where *d* is the number of columns (dimensions) of sub-arrays. The HVs are partitioned into *d*-dimensional slices and spread across different sub-arrays. As an example, Fig. 3b shows how 32 128*d*-dimensional HVs can be stored on the CAM architecture. The colors of the HV slices in Fig. 3b match where they are stored in Fig. 3a. During search, the *D*-dimensional query is also sliced into *d*-dimension slices and sent to the corresponding sub-arrays. Each sub-array outputs (i.e., votes for) the row that is most similar to the query based on the best match search demonstrated in the previous section, and sends its vote to the peripherals in the Array, which include registers and adders. The votes from Arrays are then added using the peripherals (adders and registers) in Mats. Votes from Mats are added using the peripherals (adders and registers) in Banks. Global peripherals including registers, adders, and comparators are used to add all the votes from Banks and find the row that has been voted for the most number of times, which is reported as the most similar HV to the query.

We designed a custom low-power SA (Supplementary Fig. 4) for the sub-arrays that can identify the row most similar to query by sensing the *ML* voltages of all rows. The SA latches the output of the last *ML* to discharge (i.e., the *ML* with the slowest discharge rate), and thereby indicating the best match. A detailed description of SA operation is available in Supplementary Note 1. The designed SA is agnostic of the targeted CAM precision, as well as the targeted distance function, as long as a *ML* pre-charge search approach is employed. Put another way, TCAMs with Hamming distance and MCAMs with squared Euclidean and sigmoid-like distance functions can all use the SA by (i) employing a *ML* pre-charge approach, and (ii) representing the similarity of data stored on CAM rows with the query via the discharge rate of the *ML*. Thus, identifying the *ML* with the slowest discharge, regardless of the CAM type and precision, is equivalent to finding the row that is most similar to an input query. (Note that for an MCAM with a Manhattan distance function, the *ML* voltage is too low for a *ML* pre-charge approach.)

Figure 3c–e illustrate the energy, latency, and normalized energy-delay-product (EDP) of the proposed architecture for binary, 2-bit, and 3-bit implementations (see Methods for details of evaluations). The x-axis of the heatmaps is the total number of dimensions that are supported (*D*), and the y-axis is the sub-array dimensions (*d*). The *D* values on the x-axis are multiples of 1024 (1k) and the *d* values. Figure 3c shows that energy consumption scales almost linearly when increasing *D* since we need more sub-arrays and peripherals as *D* increases. For the same *D*, larger *d* results in lower energy consumption because as *d* increases, fewer peripherals and less levels (Array, Mat, and Bank) are required as well. For example, for $D=1k$, when $d=128$ we only need one array, but when $d=16$, we need one Mat which consists of four Arrays and 32 sub-arrays. Moreover, the binary implementation on average achieves 1.24x and 1.31x energy improvement compared to 2-bit and 3-bit implementations, respectively. These improvements are associated with the higher *ML* and *DL* voltages (see Supplementary Fig. 1 for *DL* voltages) of the multi-bit implementations to ensure a squared Euclidean distance function where *ML* voltage is 0.8 V and 1 V for binary and multi-bit CAMs, respectively. The other elements of the architecture are identical and thus the energy consumption and latency of the binary and multi-bit implementations are comparable. Note that bit precision will have a significant impact on the application-level accuracy.

To understand the latency results in Fig. 3d, it is important to note that the latency of the SA scales inversely with the capacitance of the *ML*. For larger values of *d*, the capacitance of the *ML* is larger, and the *ML* discharges more slowly. For example, the SA latency for *d*=16 is 1.14 ns and for *d*=128 is 5.8ns. Moreover, all search operations happen in parallel and SA latency is constant with respect to *D*. On the other hand, the peripheral overhead (which includes interconnect) assuming smaller values of *d* is greater than when larger values of *d* are employed. Further, when *D* is sufficiently large (HVs with 6k elements), we require Bank-level peripherals regardless of *d*. Here, peripheral latency is constant and the only differentiating factor is SA latency. Thus, for larger values of *D*, *d*=16 achieves the best latency, and for smaller values of *D*, *d*=32 is best. The binary implementation on average achieves a 9% latency improvement compared to 2-bit and 3-bit implementations. This is due to the higher latency of the SA when the *ML* is pre-charged to 1 V for multi-bit implementations. Figure 3e illustrates EDP results, which can help identify design “sweet-spots”, and *d*=64 is often superior. This is explained by the fact that small values of *d* result in high energy consumption and larger values of *d* result in high latency. Obviously, the larger the *D*, the higher the EDP. However, *D* is an important parameter for application-level accuracy as will be discussed in the next section.

The proposed architecture can utilize ADCs instead of the proposed SA to perform the same operations. This can be achieved by using ADCs to digitize ML current, registers to store the outputs of the ADCs, and comparators to find the best match in each sub-array. The rest of the operations are identical to the SA-based implementation discussed above. This implementation is less expensive than work in the literature where outputs of the ADCs are aggregated across the architectural hierarchy such that final similarity results can be compared^8,13^. This is due to the overhead of moving the similarity data across the architectural hierarchy. Supplementary Fig. 5a–c show energy, latency, and EDP for 3-bit SA-based and ADC-based implementations of the proposed architecture. For each figure of merit, the heatmaps are plotted considering the same color bar. The precision of the ADCs used are *log*(*d*) to support sufficient precision for comparison of the similarities of the CAM rows and the query. Supplementary Fig. 5a shows that the SA-based implementation requires less energy than an ADC-based approach with additional peripheral overhead. The SA-based implementation is on average 4.73x more energy efficient than the ADC-based implementation. Supplementary Fig. 5b shows that the SA-based implementation is also faster than the ADC-based implementation. This is due to the fact that the SA compares 32 *ML*s simultaneously while the ADC-based implementation digitizes 32 *ML* currents and then compares them to find the best match. The SA-based implementation is on average 1.30x faster than the ADC-based implementation. Further, in terms of EDP, the SA-based implementation is preferred as shown in Supplementary Fig. 5c.

Notably, the proposed architecture can also support applications that require nearest neighbor search, which includes, but is not limited to nearest neighbor classification^31^, clustering^59^, few-shot learning^30^, class-incremental few-shot learning^60^, HDC^13^, reinforcement learning^61^, and bioinformatics^62^. The scalability of the proposed architecture allows realistic implementations of applications that need a large number of dimensions. That said, the proposed architecture (unlike other efforts^8,13^) can impact application-level accuracy due to voting-induced errors. Supplementary Fig. 6 shows a simple example of how voting can introduce errors for a binary implementation; multi-bit implementations are also prone to similar errors. In the next section, we present an end-to-end cross-layer evaluation of HDC applications using FeFET IMC compute kernels and investigate the device, circuit, and architecture non-idealities which includes the negative effects of voting in HDC applications with respect to *d* and *D*. It is noteworthy that our proposed architecture can utilize different CAM cell technologies for implementation. We evaluate a SRAM-based implementation of our architecture and compare it with FeFET-based implementations in Supplementary Fig. 7.

### In-memory HDC with FeFETs

**Figure 4:**
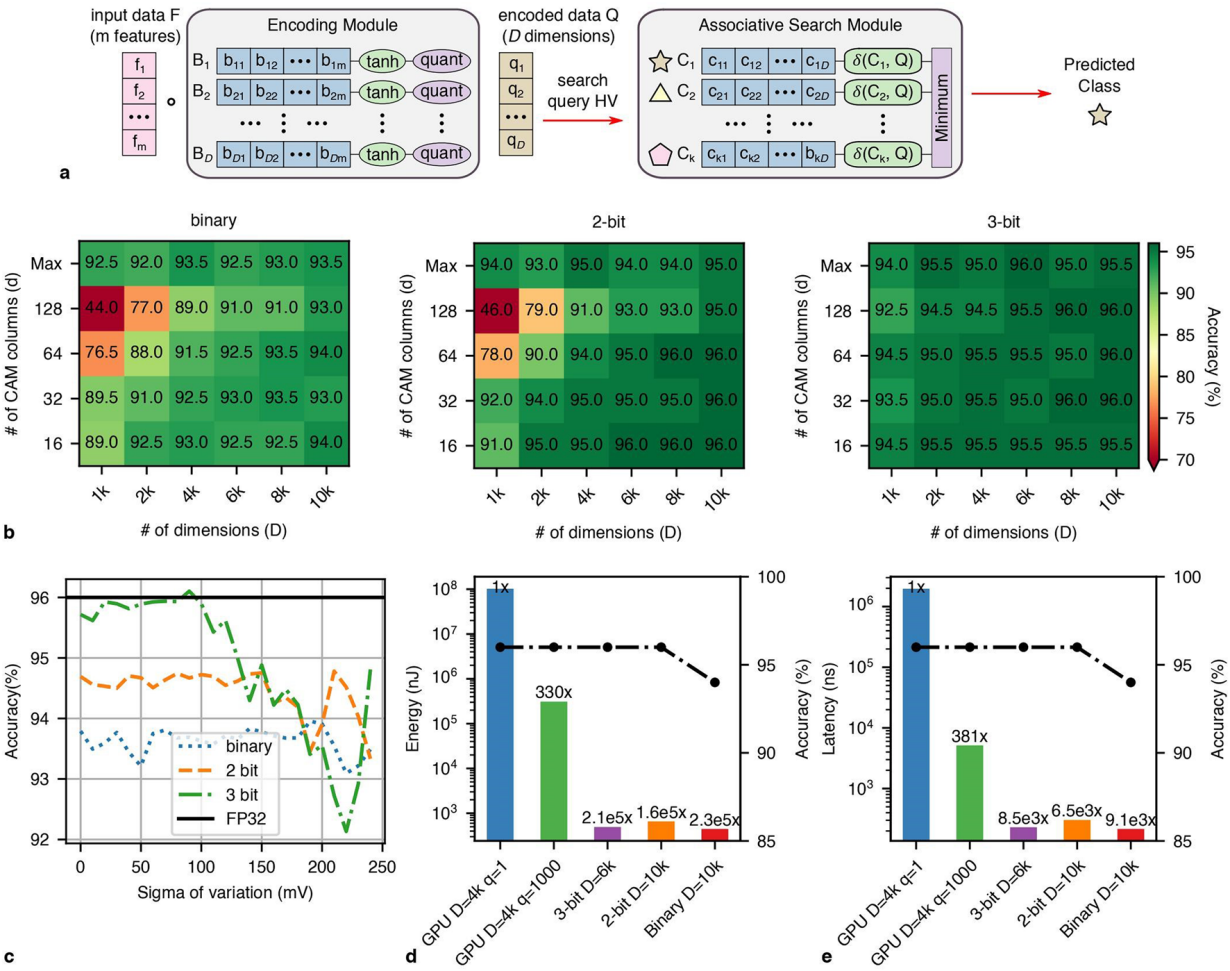
(**a**) HDC inference involves encoding the test data and searching for the most similar class HV in the associative search module. (**b**) Accuracy results for the ISOLET dataset using the proposed in-memory HDC system for different bit precisions. Similar to Fig. 3, the x-axes and y-axes are *D* and *d* of the associative search module, respectively. The heatmap plots share the same color bar where the highest accuracy is the accuracy of an FP32 4k-dimensional HDC implementation on GPU. (**c**) Effects of programming errors in terms of sigma of variation on the application-level accuracy of 5k-dimensional HDC models for the ISOLET dataset. (**d**) and (**e**) Illustrate the improvement of FeFET IMC implementations over a GPU implementation in terms of energy and latency for a single inference. The second y-axis shows the achieved accuracy of the models and the x-axis labels describe the implementation, precision, and dimensionality of the models.

HDC systems generally consist of an encoding module and an associative search module. The encoding module embeds the input data to hyperdimensional space and represents it with HVs. The encoding module is designed to map similar data (e.g., from the same class) to HVs that are similar to each other in the hyperdimensional space. The encoding module is initialized only once and is fixed during both training and inference. The associative search module stores the class HVs generated during training and finds the class HVs most similar to queries during inference. We implement a state-of-the-art non-linear encoding module design based on the Radial Basis Function (RBF) kernel^59^. Fig. 4a shows this module where base HVs ($B_i$, $1 \le i \le D$) are randomly generated from a standard normal distribution ($\mu =0,\sigma =1$) and quantized to *p* bits of precision where *p* is the precision of the HDC system. Base HVs are generated only once and are then fixed throughout training and inference. To encode input data *F* with *m* features, we compute:

1 $$\begin{aligned} Q_i = quantize(tanh(F \cdot B_i),p), 1 \le i \le D \end{aligned}$$

where the hyperbolic tangent (tanh) of the dot product of each base HV and the input data is computed. The *quantize*(., *p*) function linearly quantizes the values to *p* bits. The encoding mainly consists of MVMs that we can also perform/accelerate using FeFET crossbar arrays^21,63^. The tanh and quantization function are approximated together in hardware by taking the *p* MSB bits of the registers holding the outputs of the dot-product as the quantized values (see Supplementary Note 2). Further, unlike associative search, the computation is independent for each dimension of the query. Thus, all computation can happen in parallel using a tiled crossbar array architecture (Supplementary Fig. 9). Since MVM with crossbar arrays is a well-studied topic of research and is not the main focus of our work, we adopt the methods from the NeuroSim tool^63^ for our implementation and analysis (see Methods).

Figure 4a shows the flow of HDC inference where input data is encoded using the encoding module to a *D*-dimensional query HV. The query is then compared with the *k* class HVs ($C_i$ where $1 \le i \le k$) stored on the associative search module, with each $C_i$ representing a class. The class HV with the smallest distance (with distance function $\delta$) from the query is the predicted class. Similar to^13^, we train the HDC class HVs using software, while considering hardware constraints. To do this, we initialize the encoding module with a *p*-bit precision ($p \in \{1,2,3\}$), and use it to encode all training data. The average of all encoded data belonging to the same class *i* will be the representative class HV $C_i$, which is the starting point of the class HVs. We further improve the accuracy of class HVs with an iterative training approach^64^ while considering the underlying hardware. We keep two copies of the class HVs, the main copy and the auxiliary copy. The main copy consists of the quantized class HVs that will be stored and searched for in the associative search module during inference. The main copy uses the distance function of the associative search module (i.e., Hamming distance for binary and squared Euclidean for multi-bit) and makes predictions based on the voting method to match the inference process of the hardware. Further, the squared Euclidean distance function is based on single MCAM cell measurements (Fig. 1d). The auxiliary copy has 32-bit floating-point (FP32) precision and helps accumulate information during the iterative training process. In each iteration, training data is encoded and inference is performed. For query *Q*, if the prediction of the inference $l'$ matches the data label *l*, no updates are needed. If $l' \ne l$, class HVs $C_l$ and $C_l'$ in the auxiliary copy are updated as follows:

$$\begin{aligned} C_l \leftarrow C_l + \eta ~ (\delta _{l'}-\delta _{l}) \times Q \\ C_l' \leftarrow C_l' + \eta ~ (\delta _{l'}-\delta _{l}) \times Q \end{aligned}$$

where $\delta _{l}=\delta (C_l,Q)$, $\delta _{l'}=\delta (C_{l'},Q)$, and $\eta$ is akin to learning rate in neural network training. With this approach, the updates are proportional to the distance of the query and the class HVs. The FP32 precision of the auxiliary model ensures sufficient precision for learning. At the end of each training iteration, the main copy is updated with the quantized version of the auxiliary model. At the end of training, the main copy is stored on the proposed CAM architecture (Fig. 3a and b).

We evaluate the accuracy of the proposed solutions with widely used datasets in the HDC literature for voice recognition (ISOLET^65^), activity recognition (UCIHAR^66^ and PAMAP2^67^), and image classification (MNIST^68^ and FACE^69^). Supplementary table 3 shows the details of these datasets. Fig. 4b and Supplementary Fig. 10 show the accuracy of HDC inference for the ISOLET and other datasets using binary, 2-bit, and 3-bit FeFET implementations. The x-axes and y-axes of the plots are *D* and *d* of the associative search module, respectively. On the y-axes, $d=$Max assumes that a single CAM can store and search for *D* dimensions, which is an ideal case and is intended to show the degradation due to the voting approach. For each dataset, the three subplots share the same color bar. The lowest accuracy of the color bar is set to 70% if there are outliers. The highest accuracy on the color bar is the highest accuracy a FP32 HDC implementation on GPU can achieve (96% for ISOLET).

In Fig. 4b, $d=$Max shows that an ideal 3-bit (2-bit) implementation can achieve iso-accuracy when $D \ge 4k$, where the ideal binary implementation fails to achieve iso-accuracy even with the largest *D* ($D=10k$). Moreover, for some datasets (ISOLET, and FACE), the accuracy trends saturate when *D* is sufficiently large. This shows that accuracy improvements from continual increase in *D* will eventually be limited. The precision together with the non-linearity of the multi-bit distance function enable higher accuracies than the binary implementation given the same amount of resources. For example, the binary implementation (2-state elements) with $D=8k$ ($8k\times 2 = 16k$) has as much precision as the 3-bit implementation (8-state elements) with $D=2k$ ($2k\times 8 = 16k$), but fails to achieve similar accuracies. Further, 2-bit results are better than the binary results and often achieve iso-accuracy. Results from other datasets in Supplementary Fig. 10 follow the same trends. Binary implementations might be sufficient to achieve iso-accuracy for smaller datasets such as PAMAP2 (Supplementary Fig. 10b). However, multi-bit implementations are necessary for iso-accuracy when working on larger datasets (all other datasets studied).

HDC HVs are holographic in nature and HVs belonging to different classes are encoded to be orthogonal to each other. Thus, votes on the similarity of *d*-dimensional slices of the HVs (stored on the sub-arrays) can represent the overall similarity of the HVs. However, as illustrated in Supplementary Fig. 6, voting can have negative effects on the application-level accuracy. We study these effects by exploring *d* from 16 to 128 while also sweeping *D* from 1k to 10k. In this way we change the total number of votes (*D*/*d*), the capacity of the HVs to store information, and resilience to errors due to redundancy. Fig. 4b shows that for all implementations the accuracy loss is most significant when $d=128$ and total number of votes are small. Thus, each vote is more impactful on the final prediction. For example, when $d=128$ and $D=1k$, there are a total of 8 votes which makes an erroneous classification more probable. When *D* is smallest, accuracy decline is highest because the HVs have the lowest capacity and redundancy and are susceptible to errors. Conversely, when *D* is largest, little to no accuracy degradation is observed. Moreover, the accuracy of the binary implementation is severely degraded by the voting approach while the 3-bit implementation achieves iso-accuracy when $D \ge 6k$ with multiple *d* values across all datasets. The 2-bit implementation also loses accuracy but can often achieve iso-accuracy when $D = 10k$.

As discussed in Section "Methods", FeFET device variations can also adversely impact search accuracy and in turn application-level accuracy. Our demonstration shows that variations are expected when programming FeFETs. We study these effects by modeling the variations as Gaussian distributions^42^. Variations lead to shifts in an FeFET’s $V_t$ and can increase/decrease the cell’s current contribution to the *ML* which is the output of the cell’s distance function (Supplementary Fig. 11). Fig. 4c (Supplementary Fig. 12) shows the effects of variations on the application-level accuracy of a 5k-dimensional HDC model for the ISOLET dataset (other datasets). The x-axis is the standard deviation (sigma) of the variation and is swept from 0 mV (no variations) to 250 mV (significant variations). For each sigma of variation $\sigma$, we randomly sample values from a Gaussian distribution with a standard deviation of $\sigma$ and add it to the class HVs and then perform inference. The encoding module is instantiated randomly and is not affected by randomness in programming. We show the accuracy trends for binary and multi-bit implementations and compare it with the highest accuracy of a FP32 implementation. The binary implementation is more resilient than the multi-bit implementations due to the fact that the binary FeFETs are programmed with high and low $V_t$s and are more robust to variations. Similarly, 2-bit implementations are more robust than 3-bit implementations due to more space between target $V_t$ values. However, all implementations are quite resilient to variations such that there is no accuracy degradation for sigmas less than 100 mV for most of the datasets. The 2-bit array-level MCAM measurements in Fig. 2b show less than 100 mV sigma and support that the programming variations of current FeFET technology do not negatively impact HDC accuracy.

As discussed in Supplementary Note 1, the SA does have some limitations with respect to how accurately it can detect the distances between a query and patterns stored on a CAM. The main parameter that impacts SA accuracy is minimum detectable distance, which is defined as the minimum distance required for differentiating between the best match and the second-best match. For example, for a TCAM with a minimum detectable Hamming distance of 1, if the best match is a Hamming distance of 3 away from the query, the second-best match must be at least a Hamming distance of 4 away from the query for an accurate identification of the best match. For MCAMs, due to the complexity of the distance function, it is difficult to discuss the SA accuracy directly. Thus, for MCAMs, we report the minimum detectable distance as a percentage of the range of possible *ML* conductance. Minimum and maximum *ML* conductance are the conductance when all cells match their inputs (minimum cell conductance), and all cells observe the largest mismatch (maximum cell conductance). The largest mismatch for a *b*-bit cell occurs when it is in state 1 and its input is in state $2^b$ (or vice versa). Supplementary Fig. 13 shows how the accuracy of the SA affects the application-level accuracy. We ensure that our designed SA has a minimum detectable distance of 1 Hamming distance or 1.5% for no accuracy degradation. It is possible to relax SA accuracy for faster operation (see Supplementary Note 1), but we maintain high SA accuracy to make our CAM architecture amenable to applications that are more sensitive to such errors such as class-incremental few-shot learning^60^.

We evaluate the end-to-end energy and latency of the proposed IMC HDC system and compare our results with GPU-based implementations. An important parameter when profiling the GPU is the number of queries in a batch. More number of queries in a batch allows the GPU to parallelize the computation and improve the throughput with little to no latency overhead. Thus, we consider a single query and a thousand query batches to represent cases where batching the queries is and is not possible. Ultimately, this will be a function of the application-level use case. For example, if a real-time response is needed, batches may trend smaller; if not, larger batches may be possible. We profile the execution time of HDC inference on a GPU and show the breakdowns for encoding and associative search in Supplementary Fig. 14. Details of implementation are available in Methods. For both cases, the associative search dominates the execution time even though the encoding has more parameters than the associative search (e.g., 617x*D* vs 26x*D* for ISOLET). This is because the GPU is not as well optimized for similarity measurements as it is for MVMs. As such, the proposed CAM architecture can play a key role in IMC implementations by accelerating search.

Figure 4d and e illustrate the total energy and latency of a single inference for the ISOLET dataset (see Supplementary Fig. 15 for other datasets). We use double-axis plots to simultaneously consider accuracy in conjunction with energy and latency. Here, we consider $d=64$ and the value of *D* that leads to the highest accuracy for each bit precision for IMC implementations. The binary IMC implementation outperforms all other implementations in terms of energy and latency, but fails to achieve software-equivalent accuracies. The 2-bit IMC implementation achieves better accuracies than the binary implementation but may not reach iso-accuracy for some datasets. The 3-bit IMC implementation consistently achieves iso-accuracy at only 6k dimensions. We report results for inference of a single query on GPU at 4k dimensions for batch sizes of a single query (q=1) and a thousand queries (q=1000). As expected, the case with a thousand query batch achieves a significant improvement over the single query batch case due to the parallelism of GPU. We compare our IMC implementations with the thousand query batch case. The 3-bit IMC implementation on average reduces energy consumption and latency by 826x and 30x, respectively, when compared to a GPU implementation. Although when running on GPU, the applications are search dominant, in IMC implementations, encoding dominates energy and latency. This is due to the 8-bit precision of the inputs requiring 8 cycles to compute the output using ADCs, accumulating data from crossbars across the architectural hierarchy, and the number of features which is much larger than the number of classes. However, it is essential to accelerate both encoding and associative search to achieve maximum end-to-end improvements as the encoding can be a significant portion of the workload when running on GPU. Further, there are other applications ripe for acceleration with IMC implementations that are search dominant. It is worth noting that an IMC implementation can also achieve higher throughput by architectural-level optimizations to achieve further improvements over the GPU.

## Discussion

FeFET is a promising technology for IMC implementation. We showed that multi-bit FeFETs are not only desired, but also necessary for HDC applications with software-equivalent accuracy requirements. Both 2-bit and 3-bit FeFETs achieve higher accuracies than binary FeFETs and our 2-bit demonstration shows a path towards multi-bit FeFET IMC realizations for HDC applications. Although we did not consider the material layer in our cross-layer design analysis, material stack optimizations are highly sought after and can propel FeFETs towards higher bit-precision, lower variation, and smaller size realizations. Although, even at the current state, we showed that FeFETs are viable for IMC implementations.

In this article, we presented FeFET-based IMC systems for HDC applications that can achieve software-equivalent accuracies. We adopted a cross-layer design perspective and proposed solutions for challenges in the device, circuit, architecture, and application layers. We demonstrated, for the first time, array-level parallel FeFET MCAM operations. We proposed a scalable and efficient CAM-based architecture that can support high-dimensional associative search and is not restricted to only HDC applications. We evaluated the accuracy, energy, and latency of the proposed IMC systems for HDC applications and achieved significant energy efficiency and speedup compared to GPU implementations. We studied the effects of non-idealities from different design layers on application-level accuracy and ensured software-equivalent accuracies. The methods used in our work are extendable to other devices, circuits, architectures, and applications.

## Methods

### FeFET fabrication

The FeFET-based AND array test structures are fabricated in GlobalFoundries’ 28 nm high-k/metal gate technology node, for which co-integration with CMOS devices has been demonstrated^70^.

### FeFET MCAM array-level characterization

For the electrical characterization, passive 9$\times$7 logical-AND connected FeFET arrays are utilized. The test-structure enables direct access to row-wise connected gate-contacts along wordlines (WL) and source-/drain-contacts, connected column-wise along sourcelines (SL) and bitlines (BL). The FeFETs are characterized using a PXI-Express system from National Instruments. Each contact of the array-structures can be controlled by a NI PXIe-6570 pin parametric measurement unit (PPMU) and NI PXIe-4143 source measure unit (SMU). Source selection for each contact is handled by a custom switch-matrix, which connects to the array-structures via the probecard (Supplementary Fig. 16 and Supplementary Fig. 17). Prior to read/write operations, all FeFETs are preconditioned for 50 cycles with WL pulses of +4.5 V and -5 V at a pulse length of 500 ns each. For programming and erasing, the SMUs are utilized, keeping SLs and BLs at ground. After each program pulse, the devices are read after 300 ms to ensure sufficient time for charge detrapping. The FeFET read operation is done with the SMUs by applying a voltage ramp from 0 V to 1.4 V in 0.1 V increments while measuring current at the drain terminals, which are biased to 1.1 V. The bulk and source terminals are kept at ground. The read operation takes approximately 1 ms. To enable array-level multi-level operation a write-verify scheme is used. Initially all FeFETs of the array are set to a high $V_t$ state by a single erase pulse of -5 V for 500 ns, applied to all WLs, while having all other contacts grounded. Writing is done in parallel per WL. To mitigate device-to-device variations in the switching behavior, write voltages ranging from 1.25 V to a 5.5 V with pulse lengths of 100 ns in increments of 40 mV are applied using the SMUs. The $V_t$ of every FeFET is read after each program pulse to monitor the states. Once a FeFET reaches its target $V_t$, it is put on inhibit. The inhibit voltages are based on a VDD/3 scheme, therefore the inhibited FeFETs on the active WL are set to a SL/BL voltage of 3.2 V and the FeFETs on the passive WLs are set to a WL voltage of 1.6 V. After all FeFETs along one WL are set, writing is continued on the next WL.

The MCAMs are constructed from the AND-arrays. The BLs represent the MLs, the WLs represent the DLs, where odd WLs correspond to a DL and even WLs correspond to a $\overline{DL}$. Therefore, the 9$\times$7 arrays allow for creation of 4$\times$7 MCAMs. The WL9 is not used and the FeFETs connected to WL9 are kept in high $V_t$ state at all times to suppress leakage currents. One MCAM cell is constructed by two FeFETs that share the same BL and SL (Supplementary Fig. 2). The two FeFETs of a MCAM cell are programmed to opposing target $V_T$’s individually. For MCAM verification on array-level, a pattern (Supplementary Fig. 1) is written per WL into the AND-array by using the write-verify and inhibit schemes described above. The query search is performed with the PPMU, applying one static search condition at a time on all gate-terminals while sweeping the BLs in parallel from 0.1 V to 1.0 V in steps of 0.1 V and measuring their currents. The first query is supposed to match the stored pattern of BL1. Afterwards, 6 additional queries with increasing squared Euclidean distance are verified, as shown for BL1 in Supplementary Fig. 2. The search is repeated for all other 6 BLs, resulting in 49 search-patterns.

### FeFET MCAM single-cell characterization

For the single-cell MCAM characterization a 3-bit precision is explored. The $V_t$ states are distributed between 0.1 V to 1.15 V in steps of 150 mV. The states are set by erase operation, which is typically more gradual due to the intrinsic CPP effects^37,71^. The erase voltages range from −0.8 V to −5.0 V with pulse lengths of 100 ns in decrements of −50 mV. For the single-device characterization no inhibit-scheme is used. An MCAM cell is constructed as in the array-level characterization from two FeFETs of different WLs (Supplementary Fig. 2). To avoid leakage currents on the active BLs, the whole array is fully erased before an MCAM measurement. Afterwards, the two active WLs of the MCAMs are programmed to the lowest $V_t$. Subsequently, the setting of the two target $V_t$ states is performed by a write-verify scheme as described above, except that this time the $V_t$ state is shifted from the low $V_t$ state toward the high $V_t$ state. After a single MCAM cell is successfully programmed and read, the whole array is fully erased again, and the method can be repeated with the next two FeFETs.

### Latency and energy estimation

To evaluate and benchmark energy efficiency and latency of the proposed associative architecture, a combination of SPICE and pre-RTL simulations are employed. The Preisach FeFET model^72^ is used in HSPICE to simulate FeFET CAMs in 22 nm technology node. The Preisach model is not scalable and only supports FeFET device sizes of 200 nm by 100 nm in channel width and length. *ML* and *DL* parasitic capacitance are estimated based on the methods in^40^. The SA circuit is designed and evaluated with HSPICE assuming a 22 nm predictive technology model^73^. Adders, registers, and comparators are evaluated based on pre-RTL simulations in Aladdin^74^. Array (8 CAMs) interconnect is evaluated based on RC modeling following 22 nm design rules extracted from NVsim^75^. Flash ADCs (4 *ML*s time-share 1 ADC) are used for the ADC-based implementation and are evaluated with NeuroSim^63^. The components required for realization of the architecture with different *D* and *d* are calculated, and the total energy consumption is estimated by adding the energy consumption of all modules and a 10% global interconnect overhead as in^76^. The latency is estimated by adding the latency of components in the longest execution path, i.e., CAM and sense-amplifier latency, Array-level peripherals, Mat-level peripherals, Bank-level peripherals, and global peripherals. Again, a 10% global interconnect overhead is added. The encoding module is evaluated using NeuroSim^63^ in the 22 nm technology node. NeuroSim is an integrated framework that supports device-circuit-architecture hierarchical evaluations of crossbar designs. Supplementary Fig. 9 illustrates the tile-based encoding module. FeFET crossbar arrays of size 128$\times$128 are utilized. 8-bit inputs are applied in a bit-serial fashion to the WLs, and 4 BLs time-share a 5-bit flash ADC. The GPU energy and latency measurements are based on an NVIDIA Quadro RTX 6000 GPU built on the 16 nm process. The latency is measured for HDC applications implemented with Pytorch^77^ based on HVs with int32 datatype. Power consumption is measured using the NVIDIA System Management Interface (nvidia-smi) tool and energy consumption is calculated based on $E = Pt$.

## Supplementary Information

Supplementary Information.

## Data Availability

The data supporting plots within this paper and other findings of this study are available with reasonable requests made to the corresponding author.
