# Supplementary material for: Achieving software-equivalent accuracy for hyperdimensional computing with ferroelectric-based in-memory computing
Source: Sci Rep. 2022 Nov 10;12:19201. doi: 10.1038/s41598-022-23116-w (PMC9649759; doi:10.1038/s41598-022-23116-w)
Supplement: Supplementary file 1 — Supplementary Information. [file 41598_2022_23116_MOESM1_ESM.pdf]

Springer Nature 2021 LaTeX template

Supplemental Information

Achieving software-equivalent accuracy for hyperdimensional computing with ferroelectric-based in-memory computing

Contents

Supplementary figures

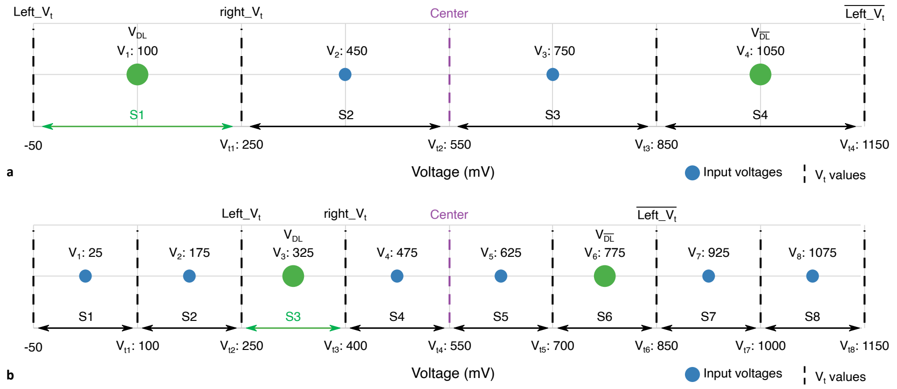

Supplementary Figure 1 V_t and V_{DL} values for (a) 2-bit and (b) 3-bit implementations of 2FeFET MCAM.

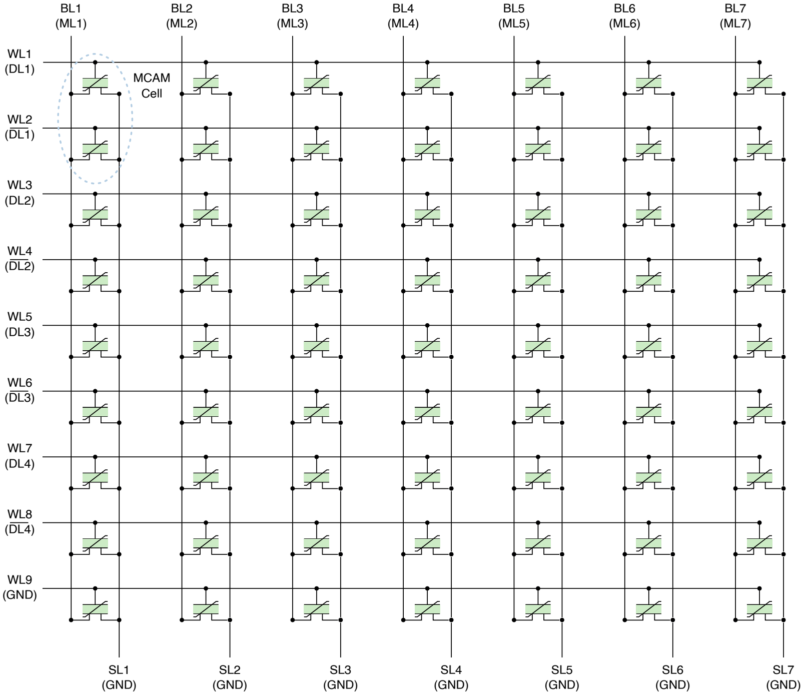

Supplementary Figure 2 FeFETs arranged in a 9×7 AND array structure are used to demonstrate a 7 row and 4 column MCAM. The bitlines (BL) are the MLs, the WLs are the DLs, and the SLs are connected to the ground (GND). WL9 is grounded and all the FeFETs connected to it are in high V_t state as there are not enough WLs to support another MCAM column.

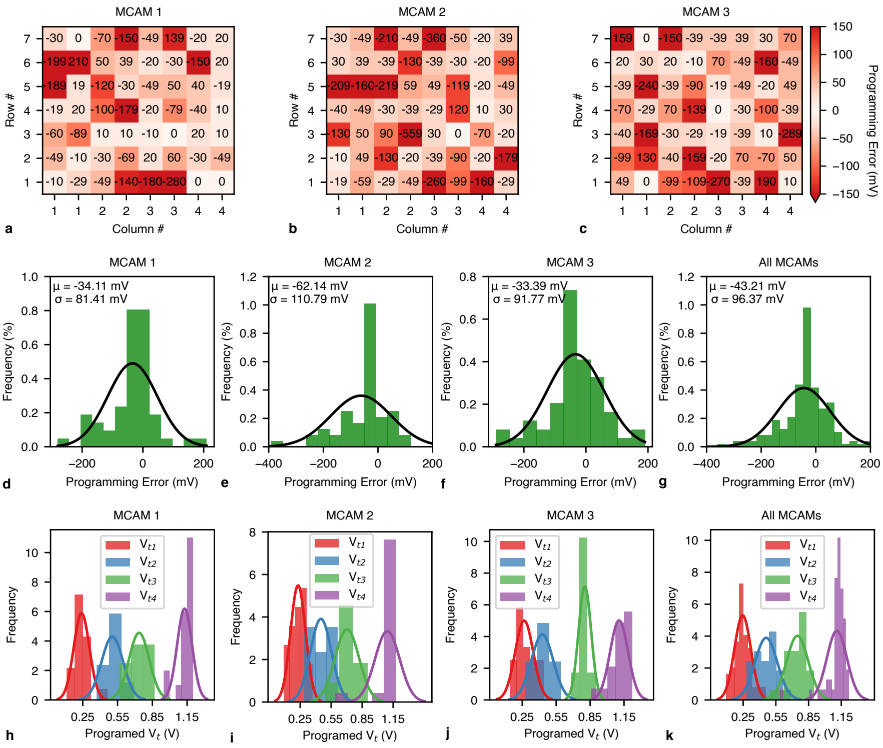

Supplementary Figure 3 (a), (b), and (c) show the difference between the programmed V_t and the target V_t (programming error) for the three MCAMs with respect to the targets in Supplementary Table 1. (d), (e), (f), and (g) show the distribution of the programming errors for the three MCAMs and all MCAMs combined. (h), (i), (j), and (k) show the distribution of programming errors with respect to different target V_ts for the three MCAMs and all MCAMs combined.

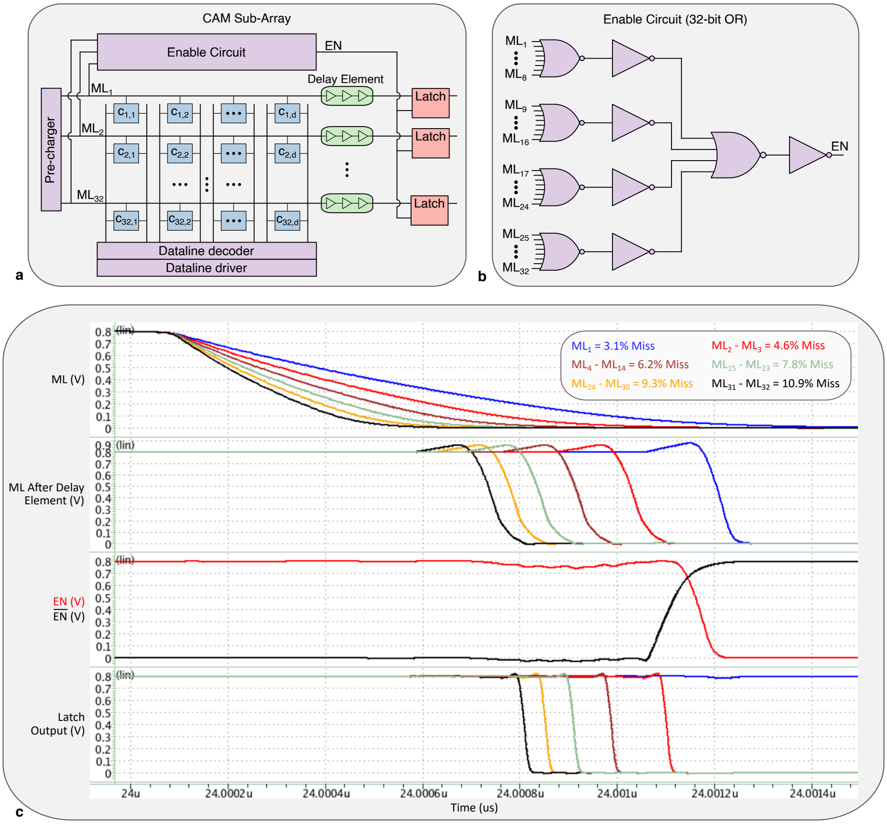

Supplementary Figure 4 (a) shows a CAM sub-array with the designed SA. The SA consists of an enable circuit, delay elements, and latches. (b) the enable circuit is a 32-bit OR gate implemented using four 8-bit OR gates. (c) shows the simulation waveforms for the designed SA. The signals depicted are ML voltage, ML voltage after the delay elements, enable signals EN and $\overline{EN}$, and the outputs of the latches. The mismatch percentage between the query and the data stored on different MLs is noted on the top right and is color coded. The SA is able to correctly identify the ML with the lowest discharge rate, i.e., $ML1$.

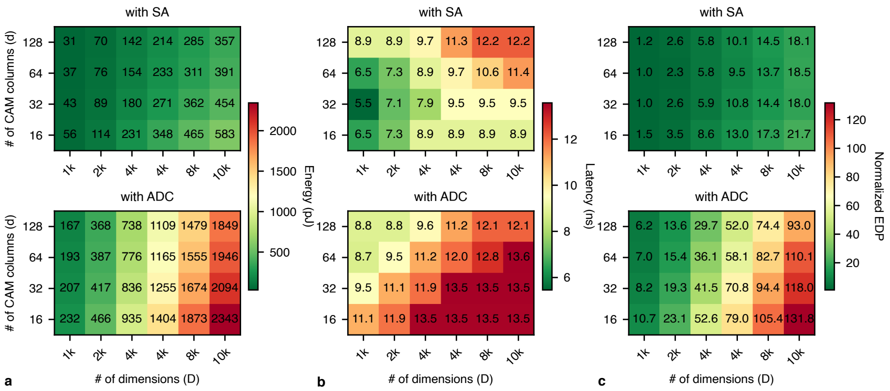

Supplementary Figure 5 (a) energy, (b) latency, and (c) normalized EDP of a single search with the proposed architecture for 3-bit SA-based and ADC-based implementations.

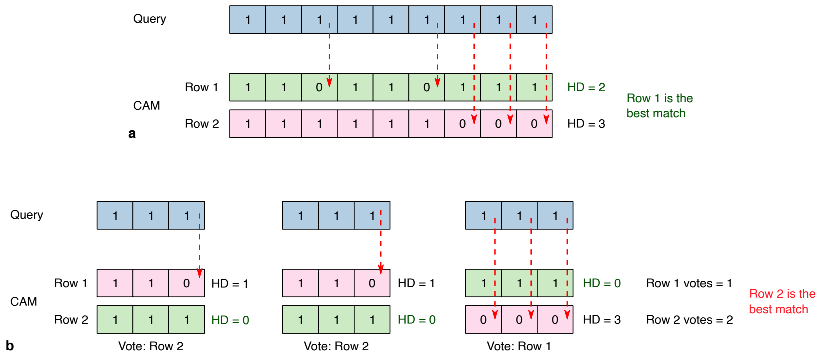

Supplementary Figure 6 (a) shows a simple example of best match search for a 9-dimensional binary CAM. The query is searched in a CAM with 2 rows. Row 1 is the best match since its Hamming distance (HD) with the query is only 2 while the HD of row 2 with the query is 3. (b) shows the same query and CAM patterns split between three 3-dimensional CAMs following our proposed method in Fig. 3. Row 2 gets 2 votes as best match and Row 1 gets 1 vote as best match, and Row 2 is incorrectly reported as the best match due to the voting method. Similar errors are possible for multi-bit CAMs as well.

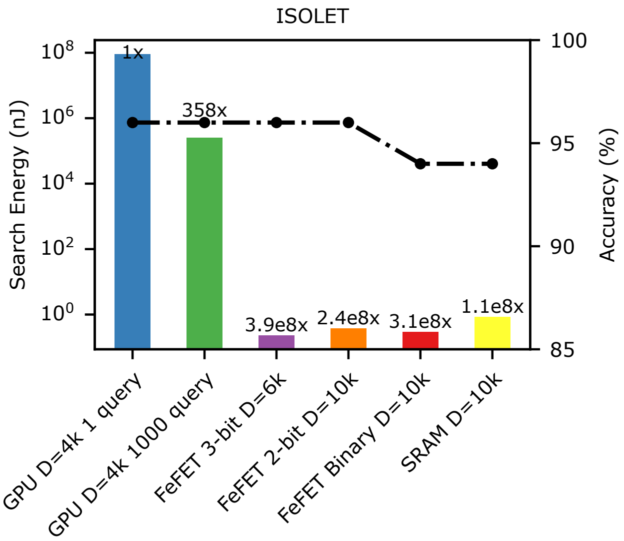

Supplementary Figure 7 Search energy results for the ISOLET dataset. The second y-axis shows the accuracy of each implementation. The energy results are for a single inference. The SRAM data point is for a SRAM-based implementation of our architecture. 3-bit FeFET and binary FeFET achieve 3.5× and 2.8× improvement over the SRAM-based implementation, respectively.

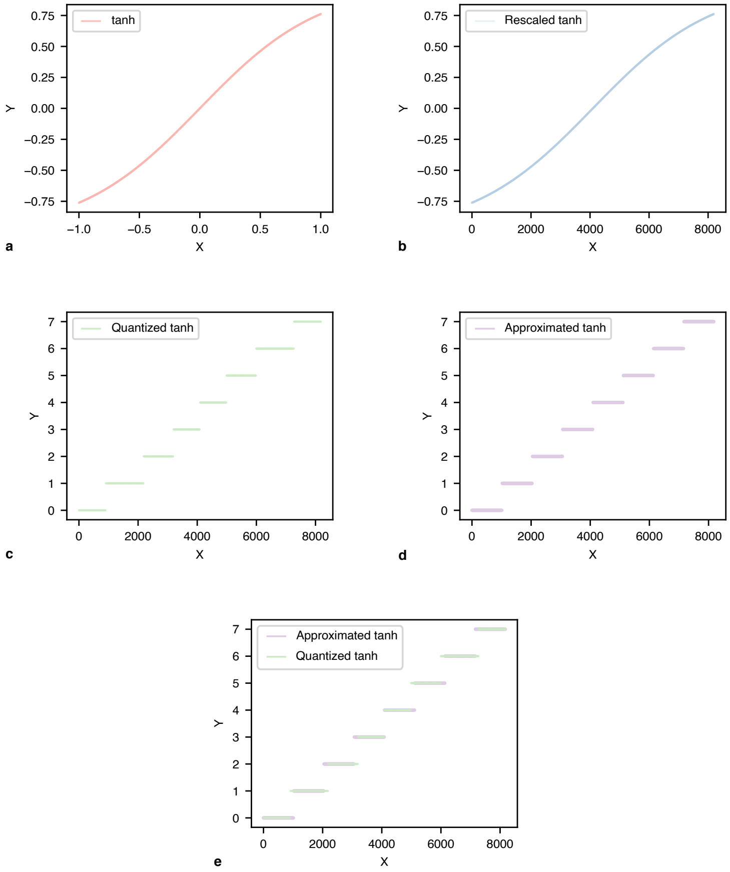

Supplementary Figure 8 (a) tanh function for inputs between [-1, 1], (b) tanh rescaled for 13-bit inputs, (c) rescaled tanh quantized to 3 bits, (d) the approximated tanh using MSBs, and (e) the difference between the approximated tanh and quantized tanh.

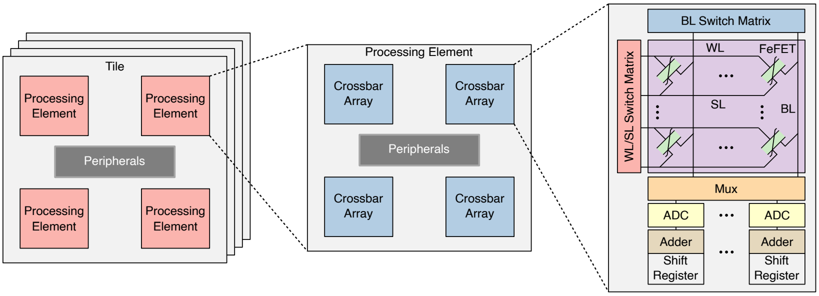

Supplementary Figure 9 Scalable tiled architecture based on FeFET crossbars used to implement encoding.

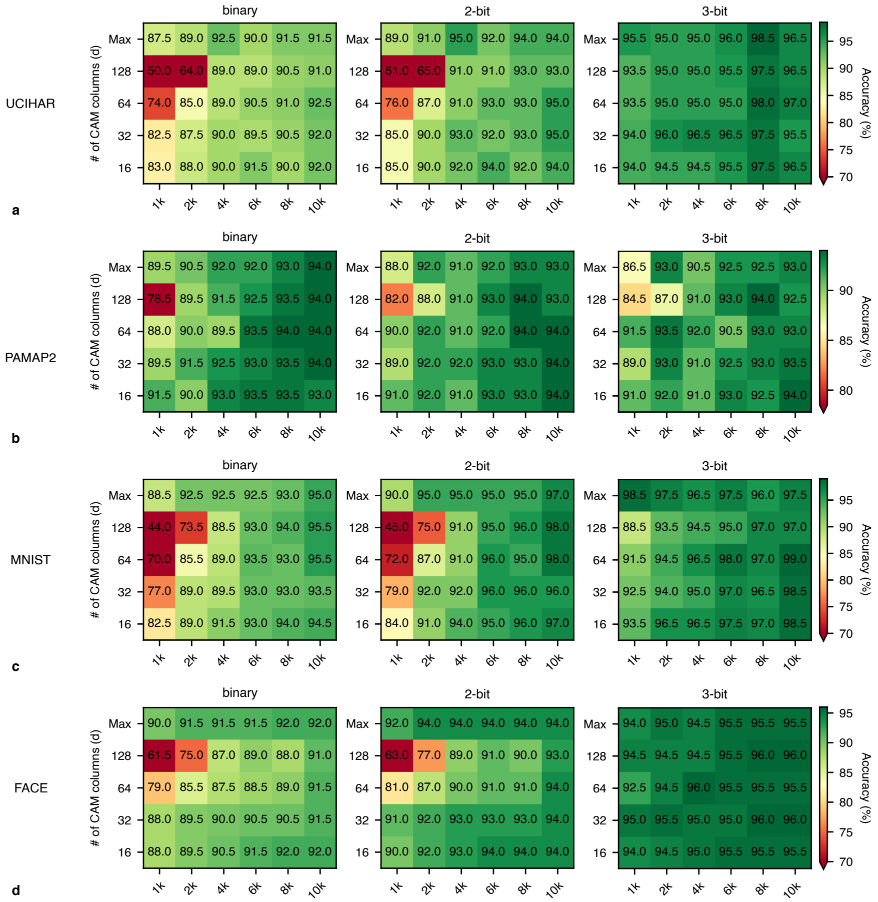

Supplementary Figure 10 Accuracy results for (a) UCIHAR, (b) PAMAP2, (c) MNIST, and (d) FACE datasets. Similar to the figures in the main text, the x-axes and y-axes are D and d of the associative search module, respectively. The heatmaps share the same color bar where the highest accuracy is the accuracy of a FP32 4k-dimensional HDC implementation on GPU.

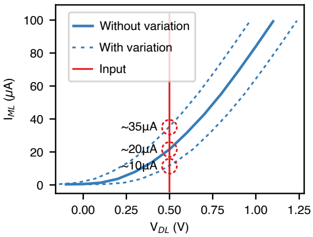

Supplementary Figure 11 FeFET variations shift the V_t of the devices and can change I_{ML} which is the distance function.

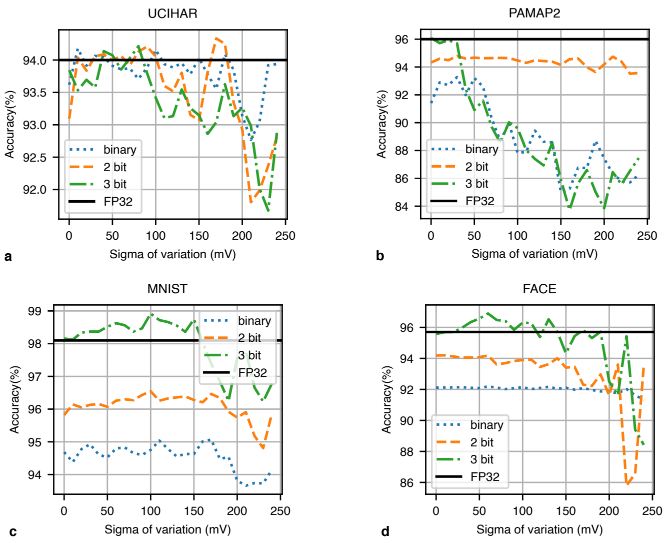

Supplementary Figure 12 Accuracy results for a 5k-dimensional HDC model for (a) UCIHAR, (b) PAMAP2, (c) MNIST, and (d) FACE datasets. The x-axis is the standard deviation of the Gaussian sampled for variations.

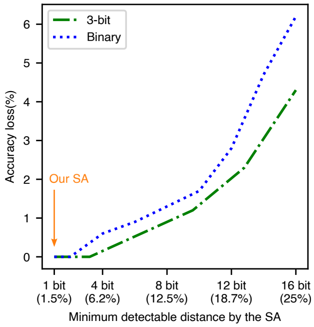

Supplementary Figure 13 The effects of minimum detectable distance of the SA for binary and 3-bit implementations in terms of number of bits and distance percentage. The results are for 4k-dimensional implementations using 64 column CAMs (D=4k and d=64).

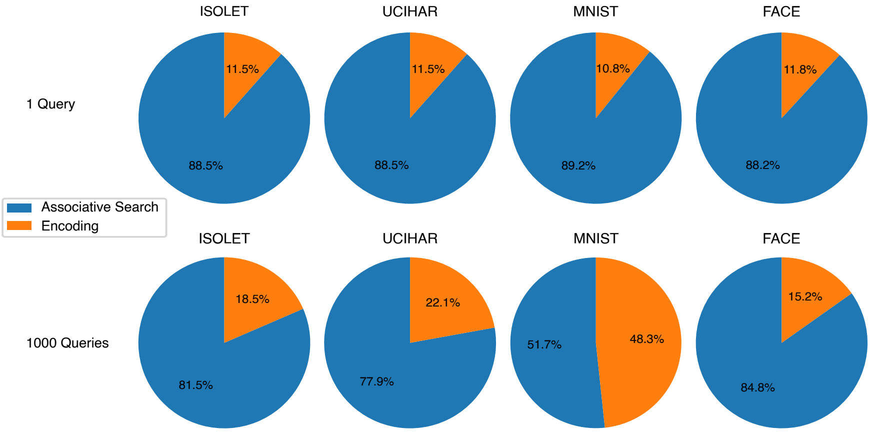

Supplementary Figure 14 The associative search and encoding breakdown of GPU execution time for different datasets. We consider two cases with a single query and a thousand queries.

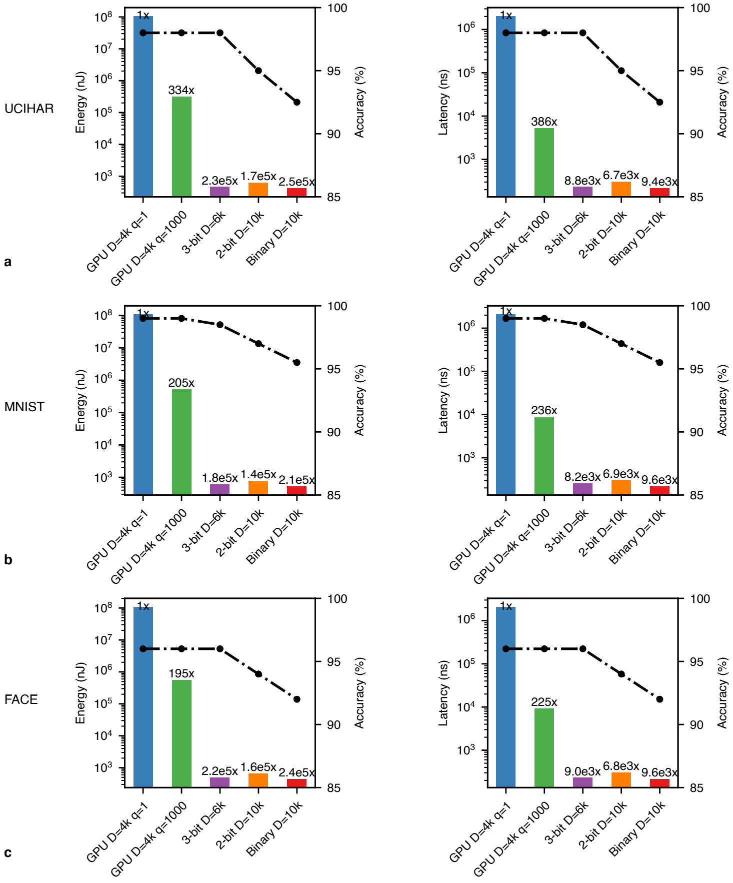

Supplementary Figure 15 Energy and latency results for (a) UCIHAR, (b) PAMAP2, (c) MNIST, and (d) FACE datasets. The second y-axis shows the accuracy of each data point. The results are for a single inference.

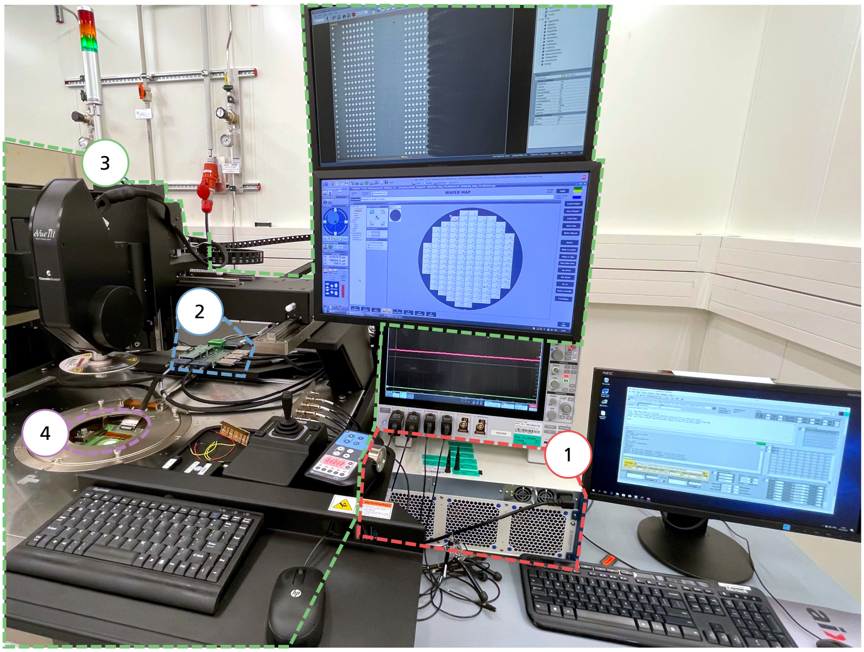

Supplementary Figure 16 Measurement setup including (1) PXI-System providing the SMUs and PPMUs, (2) Custom Switch-Matrix for source-selection, (3) semi-automatic probe-station, and (4) Probe-card connection to wafer.

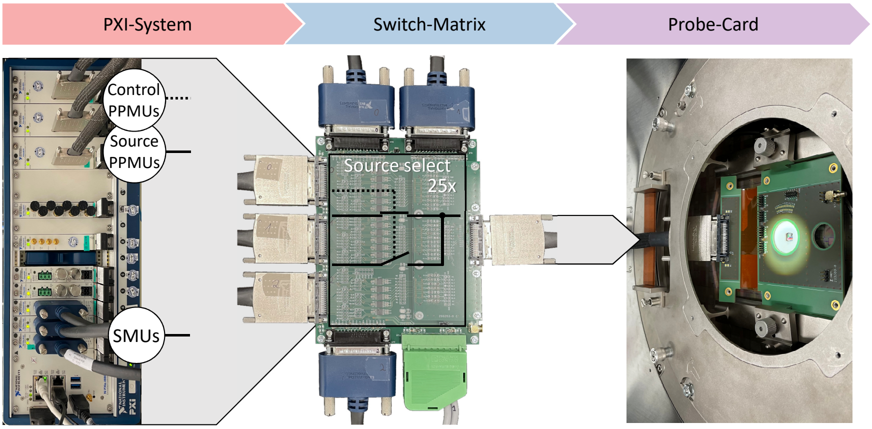

Supplementary Figure 17 Flow-chart summarizing the AND-array-compatible measurement setup.

Supplementary tables

Supplementary Table 1 Patterns stored on the MCAMs and the target V_t values for $left\_V_t$ (l_V_t) and $right\_V_t$ (r_V_t)

ML #	d1	d2	d3	d4	l_V_t1 (V)	r_V_t1 (V)	l_V_t2 (V)	r_V_t2 (V)	l_V_t3 (V)	r_V_t3 (V)	l_V_t4 (V)	r_V_t4 (V)
1	0	3	1	0	0.25	1.15	1.15	0.25	0.55	0.85	0.25	1.15
2	3	0	3	1	1.15	0.25	0.25	1.15	1.15	0.25	0.55	0.85
3	1	0	0	0	0.55	0.85	0.25	1.15	0.25	1.15	0.25	1.15
4	0	0	3	0	0.25	1.15	0.25	1.15	1.15	0.25	0.25	1.15
5	2	2	0	3	0.85	0.55	0.85	0.55	0.25	1.15	1.15	0.25
6	3	3	3	1	1.15	0.25	1.15	0.25	1.15	0.25	0.55	0.85
7	0	1	2	3	0.25	1.15	0.55	0.85	0.85	0.55	1.15	0.25

Supplementary Table 2 Queries searched on the MCAM, V_{DL} and $V_{\overline{DL}}$, and squared Euclidean distance from the best match pattern ($dist^2$)

$dist^2$	d1	d2	d3	d4	$V_{DL}1$ (V)	$V_{\overline{DL}}1$ (V)	$V_{DL}2$ (V)	$V_{\overline{DL}}2$ (V)	$V_{DL}3$ (V)	$V_{\overline{DL}}3$ (V)	$V_{DL}4$ (V)	$V_{\overline{DL}}4$ (V)
0	0	3	1	0	0.1	1	1	0.1	0.4	0.7	0.1	1
1	0	2	1	0	0.1	1	0.7	0.4	0.4	0.7	0.1	1
1	1	3	1	0	0.4	0.7	1	0.1	0.4	0.7	0.1	1
2	1	2	1	0	0.4	0.7	0.7	0.4	0.4	0.7	0.1	1
2	0	2	0	0	0.1	1	0.7	0.4	0.1	1	0.1	1
3	1	3	0	1	0.4	0.7	1	0.1	0.1	1	0.4	0.7
3	0	2	0	1	0.1	1	0.7	0.4	0.1	1	0.4	0.7

Supplementary Table 3 HDC datasets

Dataset	# of features	# of classes	# of train samples	# of test samples	Description
ISOLET	617	26	6,238	1,559	Voice Recognition [65]
UCIHAR	561	12	6,213	1,554	Activity Recognition [78]
PAMAP2	75	5	611,142	101,582	Activity Recognition [67]
MNIST	784	10	60,000	10,000	Digit Recognition [79]
FACE	608	2	522,441	2,494	Face Recognition [69]

Supplementary Table 4 Maximum detectable distance of the designed SA

# of CAM columns	max detectable conductance (S)	max detectable Hamming distance
16	6	26
32	8	12
64	10	5
128	12	10

Supplementary Notes

Supplementary Note 1: Best match sense amplifier

Supplementary Fig. 4a shows the designed SA for detecting the best match in a CAM sub-array. The SA includes an enable circuit, delay elements, and latches. The enable circuit (Supplementary Fig. 4b) is a 32-bit OR gate and detects when the last ML voltage goes low. For searching the CAM sub-array, the MLs are pre-charged to a high voltage (1 V for MCAM and 0.8 V for TCAM), the EN signal is '1', and all the latches constantly sample the ML voltage after the delay elements. After the pre-charge phase, the input voltages are applied to the DLs, and the ML voltages start to drop. The EN signal is '1' until all the MLs drop low and then the EN signal changes to '0'. When the EN signal goes to '0', the latches stop sampling the ML and keep the last voltage they sampled. The delay elements ensure that when the latches last sample the ML voltage, it is still high. To illustrate the operation of the proposed SA we provide examples with TCAMs to simplify the discussion. Supplementary Fig. 4c shows waveforms of SPICE simulations for best match search with the designed SA. The Hamming distance between the query and the MLs are shown on the left of the figure. The signals shown are ML voltage, ML voltage after the delay elements, EN voltage, and latch output. $ML1$ is the last ML that drops low (4 Hamming distance) and the EN signal goes to '0' when $ML1$ goes low. The latch outputs show that the SA successfully detects that $ML1$ is the best match.

The designed SA works based on the RC discharge mechanisms of the MLs and has limitations with respect to how accurately it can detect the RC differences. Specifically, when the discharge rates of the best match and the second best match are too close, the SA may report two rows as match. The two main parameters that affect the accuracy of the SA are: (i) the number of CAM columns which affects the capacitance (C) of the MLs and (ii) the distance between patterns stored on the MLs and the query which affects the ML conductance (or resistance (R)). We tune the delay elements in the SA such that for different number of CAM columns the minimum detectable distance between the best match and the second best match (referred to as minimum detectable distance) is 1 Hamming distance which is equivalent to 1.5% distance. To maintain this level of sensing accuracy for more number of columns, longer time is spent. The latency of the SA for a TCAM with 16, 32, 64, and 128 columns is 0.86 ns, 1.2 ns, 2.6 ns, and 4.2 ns, respectively. For MCAMs, the ML voltage is higher and the SA latency for 16, 32, 64, and 128 columns is 1.14 ns, 1.69 ns, 3.5 ns, and 5.8 ns, respectively. It is worth noting that given application-level requirements and limits, it is possible to reduce the precision of the SA for faster operation. Supplementary Fig. 13 shows that for a 3-bit MCAM, a minimum detectable distance of 5% is sufficient for HDC applications. However, we report SA numbers for 1 bit (1.5%) minimum detectable distance as our proposed architecture is general and amenable to many applications.

Another parameter that affects the accuracy of the SA is the distance of the best match from a query (d_{bm}). When d_{bm} is small, e.g., 1 or 2 Hamming distances, the ML discharge is slow and it is easier for the SA to detect it. On the other hand, when d_{bm} is larger, e.g., 9 or 10 Hamming distances, the ML discharge is fast and can hinder correct best match detection. The designed SA can only detect the best match when $d_{bm} \leq 9$ Hamming distance, with 1 minimum detectable Hamming distance. When $d_{bm} = 10$ Hamming distance, the minimum detectable Hamming distance is 2. This does not pose a problem for HDC applications studied in this work since the best match is almost always more than 90% similar to the query, regardless of bit precision. That means $d_{bm} < 7$ Hamming distance. This analysis applies to the 2-bit and 3-bit MCAMs as well since the SA uses the same mechanism regardless of bit precision.

Supplementary Note 2: Quantized tanh implementation in hardware

The tanh function in the encoding module (Fig. 4a) is intended to rescale and clip the outputs of the MVM. In the software FP32 implementation, the outputs of the MVM are first normalized. Supplementary Fig. 8a shows the tanh function for values within the range (-1, 1) which matches the normalized values of the MVM. However, in our hardware implementation, the outputs of the MVM are non-negative. The inputs have 8-bit precision and are applied to the crossbar arrays one bit at a time. The ADCs used to convert current to digits have 5-bit precision. The outputs of the ADCs are shifted and added using shift registers and adders. Thus, the outputs of the MVM have 13 bits of precision. Supplementary Fig. 8b shows tanh rescaled to a 13-bit input range. The quantized tanh function converts the 13-bit values to p bits. Supplementary Fig. 8c shows a quantized tanh function scaled for values within range (0, 2^{13}) where the outputs have 3-bits of precision. To approximate this in hardware, we take the p MSB bits of the MVM outputs as the output of the quantized tanh function (Supplementary Fig. 8d). The approximated tanh is plotted with the quantized tanh in Supplementary Fig. 8e and shows that it approximates the behavior of the quantized tanh quite well. We implement this approximated tanh for our accuracy evaluations. Approximate tanh has no effects on the end-to-end application-level accuracy of our HDC case studies.
